# Exploration of PDCoV-induced apoptosis through mitochondrial dynamics imbalance and the antagonistic effect of SeNPs

**DOI:** 10.3389/fimmu.2022.972499

**Published:** 2022-08-23

**Authors:** Zhihua Ren, Yueru Yu, Xiaojie Zhang, Qiuxiang Wang, Junliang Deng, Chaoxi Chen, Riyi Shi, Zhanyong Wei, Hui Hu

**Affiliations:** ^1^ College of Veterinary Medicine, Henan Agricultural University, Zhengzhou, China; ^2^ Key Laboratory of Animal Disease and Human Health of Sichuan Province, College of Veterinary Medicine, Sichuan Agricultural University, Chengdu, China; ^3^ College of Animal and Veterinary Sciences, Southwest Minzu University, Chengdu, China; ^4^ Department of Basic Medical Sciences, College of Veterinary Medicine, Weldon School of Biomedical Engineering, Purdue University, West Lafayette, IN, United States

**Keywords:** Porcine Deltacoronavirus, selenium nano-particles, apoptosis, mitochondrial dynamics, swine testicular cells

## Abstract

Porcine Deltacoronavirus (PDCoV), an enveloped positive-strand RNA virus that causes respiratory and gastrointestinal diseases, is widely spread worldwide, but there is no effective drug or vaccine against it. This study investigated the optimal Selenium Nano-Particles (SeNPs) addition concentration (2 - 10 μg/mL) and the mechanism of PDCoV effect on ST (Swine Testis) cell apoptosis, the antagonistic effect of SeNPs on PDCoV. The results indicated that 4 μg/mL SeNPs significantly decreased PDCoV replication on ST cells. SeNPs relieved PDCoV-induced mitochondrial division and antagonized PDCoV-induced apoptosis *via* decreasing Cyt C release and Caspase 9 and Caspase 3 activation. The above results provided an idea and experimental basis associated with anti-PDCoV drug development and clinical use.

## Introduction

Porcine Deltacoronavirus (PDCoV), an enveloped RNA virus, is a swine gastrointestinal pathogenic virus. Infection with the virus causes diarrhea, dehydration, and vomiting in sows and piglets (1–3). PDCoV was first detected in Hong Kong, China, in 2012 (4) and has spread to many regions and countries worldwide (5–10), causing colossal impact and economic losses to the global swine industry. In recent years, many studies have reported the isolation of PDCoV from other species, such as cattle and poultry (11–13). In addition to infecting animals, researchers identified PDCoV, which come from two distinct viral lineages in plasma samples of three Haitian children with acute undifferentiated febrile illness (14). These indicate that PDCoV has the capability of cross-species transmissions, which deserve high priority. Furthermore, studies have reported that added trypsin could increase virus titer, accompanied by visible CPE compared to no or lower trypsin concentrations (15, 16).

Apoptosis is programmed cell death that reduces the size of apoptotic cells, chromatin consolidation in the nucleus, and degradation and fragmentation of DNA under physiological or pathological conditions (17, 18). PDCoV infection induces tissue or cell apoptosis. Duan et al. detected a great number of apoptotic signals in the jejunum and ileum of pigs infected with the PDCoV strain CHN-HN-1601 by oral administration with 1 × 106 TCID_50_ (19). The experiment of Jung et al. showed that piglets inoculated orally with 8.8 - 11.0 log_10_ genomic equivalents (GE) of US PDCoV strain OH-FD22 do not induce apoptosis in gut cells but induce LLC-PK and ST cells apoptosis *in vitro (* 1). Therefore, the effect of this virus on apoptosis deserves further study.

Cytochrome C (Cyt C) is a crucial apoptotic signaling molecule, and its release from mitochondria is a key event in apoptosis (20). Cyt C released into the cytoplasm binds to Apoptosis protease activating factor-1 (Apaf-1) to form an apoptotic complex, which activates Caspase 9 and Caspase 3 to activate the apoptotic cascade pathway and ultimately induce apoptosis (21, 22). Mitochondria are important organelles in cells. Many studies have shown that the abnormal function of mitochondria is closely related to the occurrence of apoptosis. According to previous studies, approximately 15% of Cyt C is bound to the outer and inner membrane of the mitochondrial membrane gap in a normal physiological environment. Still, this binding is not tight and is easily separated by external stimuli. The remaining 85% of Cyt C is located in the mitochondrial cristae (23, 24), and Cyt C release is associated with mitochondrial dynamics homeostasis and its associated proteins. Mitochondrial dynamics is an important event that affects the morphology and number of mitochondrial networks; this process is regulated by mitochondrial fission proteins (Drp1, Fis1) and mitochondrial fusion proteins (Mfn1, Mfn2, and OPA1); when the dynamic balance of mitochondria is disrupted, it can lead to changes in mitochondrial morphology and number or structural damage, which affects many biological processes, including apoptosis (22).

Selenium is a trace element that is essential in the body and inhibits the replication of many viruses, making it an excellent candidate for treating viral infections. According to research, selenium-deficient mice are more susceptible to Coxsackie virus B (CVB) and influenza virus (IV), as well as having more severe organ and tissue damage (25, 26). Furthermore, the cure rates of the new coronavirus (SARS-CoV-2) is strongly related to selenium intake and status in different Chinese areas (27). Serum selenium levels are linked to the prognosis of Corona Virus Disease 2019 (COVID-19), and higher serum selenium levels are related to a higher cure rate (28). In a previous study by our group, it was discovered that Se-Met inhibited PDCoV replication *in vitro*, and selenium could alleviate the viral infection-induced oxidative stress, and increased the levels of various cytokines in host cells, boosting the level of cellular immunity to inhibit virus replication (29). To sum up, selenium may be a promising drug for treating COVID-19 and other virus infections, allowing for the development of new and highly effective antiviral drugs. Furthermore, Selenium Nano-Particles (SeNPs) are characterized by high activity, low toxicity, and easy absorption by the human body, and exhibit good antiviral activity against dengue virus (DENV) in Hela and HepG2 cells (30). SeNPs has powerful antioxidant and cytotoxicity-reducing properties (31). On the one hand, SeNPs can promote apoptosis of cancer cells *in vitro* through the mitochondrial pathway (32), and on the other hand, they have antioxidant, anti-oxidative stress, and anti-apoptotic effects on focal tissues (33). In addition, SeNPs can reduce mitochondrial dysfunction in disease states, thereby reducing apoptosis (34).

We cultured swine testis (ST) cells *in vitro*, performed and compared the optimal trypsin addition concentration in PDCoV infection, and investigated the potential relationship between mitochondrial homeostasis-related proteins and apoptosis and apoptotic proteins under PDCoV infection, as well as the effect of SeNPs addition on virus replication and apoptosis, providing a theoretical foundation for drug research and development and clinical medication against PDCoV.

## Materials and methods

### Cells and virus

Porcine testicular cells were cultured with 90% DMEM high sugar medium, 9% fetal bovine serum, 1% Penicillin-Streptomycin-Mycoplasma Removal Agent Solution, incubated at 37°C. The PDCoV HNZK-04 strain (GenBank accession no: MH708124.1) isolated and preserved according to the method of Jin (35) et al. was used in this experiment. ST cells were infected with PDCoV (MOI=0.07).

### Drugs

SeNPs were purchased from Yantai Jialong Nano Industry Co., Ltd., lot 202107027, 100g, red liquid. Trypsin (powder) was purchased from Sigma Aldrich.

### Characterisation and identification of SeNPs

SeNPs were characterized using transmission electron microscopy (TEM) and Energy Dispersive X-ray (EDX). TEM was used to observe the morphological structure of the SeNPs, including shape and size, and EDX was used to identify some of the elements. The experimental characterization was carried out at the Fuda testing group in Shanghai.

### CCK-8 assay for cytotoxicity of trypsin and SeNPs

The CCK-8 method was used to assess the cytotoxicity of trypsin and SeNPs *in vitro*. A control group (cells and culture medium) and a test group (cell culture medium, trypsin, or SeNPs) were set up. In 96-well plates, cells were seeded and incubated overnight. After two D-Hanks washes, trypsin was added at 2, 3, 4, 5, 6, 7, 8, 9, and 10 μg/mL (n=6). After washing the cells twice with D-Hanks solution, CCK-8 solution was added, and absorbance at 450 nm was measured using an enzyme marker. After pre-experimentation, SeNPs were added to the culture medium at final concentrations of 0, 1, 2, 4, 6, 8, 10, 12, 14 μg/mL. The preceding experimental steps were followed.

### The detection of viral replication and mitochondrial dynamics related-protein mRNA expression by RT-qPCR

Total cellular RNA was extracted using the traditional Trizol method. cDNA was synthesized and amplified using the TransScript All-in-One First-Strand cDNA Synthesis SuperMix for the qPCR kit, and cDNA was quantified in the CFX Connect real-time PCR detection system (BIO-RAD). Relative gene expression levels were calculated using Equation 2^-(ΔΔCt)^ (using β-actin as the reference gene). Primers were designed according to the NCBI database, and Primer Premier 5 software and the primer sequences shown in **Table 1** were pre-experimentally screened.

**Table 1:** Primer sequence.

Gene name	Sequence (5’-3’)
*Mpro*	F: CTTATTCTGCTTTGGCTGCTC
	R: GGATATGAAGGTTAGTACGGC
*β-actin*	F: GGCACCACACCTTCTACAACGAG
	R: TCATCTTCTCACGGTTGGCTTTGG
*Drp1*	F: AATTGAGGCCGAGACAGACC
	R: GGAACTCGATGTCAGGAGGC
*Fis1*	F: ACAGAGCCACAGAACAACC
	R: AGTCCAATGAGTCCAGCC
*Mfn1*	F: AAGGAACGGATGGAGATAAAGC
	R: TGCGACCAAAACGAAGACATC
*Mfn2*	F: GGGCATTCTCGTTGTTGG
	R: AGCTTCTCGCTGGCGTACT
*OPA1*	F: CGAAAGAACCTTGAATCCC
	R: AATAGAAGCCTCTCCGACA

The experiment “The effect of trypsin on PDCoV-infected ST cells” was divided into two groups: virus (respectively added 1, 2, 3 and 4 μg/mL trypsin) and control and PDCoV replication were measured 48 h after viral infection (n=6).

The experiment “The mRNA expression of mitochondrial dynamics-related proteins in ST cells after PDCoV infection” was divided into two groups: virus and control, and RT-qPCR assays were performed at 6, 24, and 48 h after PDCoV infection (n=6).

The experiment “The inhibitory effect of SeNPs on PDCoV replication” was divided into four groups: virus, virus plus 2μg/mL SeNPs, virus plus 4μg/mL SeNPs and virus plus 8μg/mL SeNPs. RT-qPCR was used to assess intracellular viral replication 6, 24, and 48 h after PDCoV infection (n=6).

### Infectivity of PDCoV on ST cells at different trypsin concentrations by indirect immunofluorescence assay

The cells were seeded with cell slides, and after culturing for a certain time, the slides were stained, and the fluorescence was observed using an Olympus inverted biological microscope.

The test was grouped into control and viral groups and tested at 48 h post-infection.

### Western blotting detects the expression of ST cell apoptosis-related proteins and mitochondrial dynamics-related proteins after PDCoV infection

Total Protein: Cellular proteins were extracted according to the extraction kit, and concentrations were determined using the BCA Protein Concentration Assay Kit. Mitochondrial proteins: the cell pellet was collected after the cells were digested, triturated 30 - 40 times on ice with a homogenizer added to HEPES solution, and after multiple centrifugal cleavages, the supernatant was collected to determine the protein concentration using a BCA protein concentration assay kit.

The experiment “Effect of SeNPs on the expression of mitochondrial dynamics-related proteins in ST cells after PDCoV infection” was grouped into virus ,control, SeNPs, and virus plus SeNPs groups, and WB assays were performed at 6, 24, and 48 h (n=3).

The experiment “Effect of SeNPs on the expression of apoptosis-related proteins in PDCoV-infected cells” was divided into four groups: control, virus, SeNPs, and virus plus SeNPs groups, with WB assay at 6, 24, and 48 h (n=3).

### Flow Cytometry- Annexin-FITC/PI double-staining assay for apoptosis detection

Cells were stained according to the Annexin V-FITC Apoptosis Detection Kit (Wuhan servicebio Technology Co., Ltd.), followed by 100 - 200 μL cell suspension for apoptosis detection using flow cytometry FITC and PE passages, with blank and isotype control tubes first, followed by tubes to be tested. Flowjo V10 software was used to analyze the results.

The test “Inhibition of PDCoV-induced ST cell apoptosis by SeNPs” was divided into control, virus, SeNPs, and virus plus SeNPs groups and assayed by flow cytometry at 6, 24, and 48 h (n=3).

### Statistical analysis

The test results are expressed as mean ± standard deviation. The experimental data were sorted and unified in Excel, and SPSS 21.0 software was used for significant difference analysis, and the least significant difference (LSD) method was used to compare the data, respectively. *P* < 0.05 indicates statistical significance.

## Results

### Optimal trypsin concentration selected under PDCoV infection

**Figure 1:**
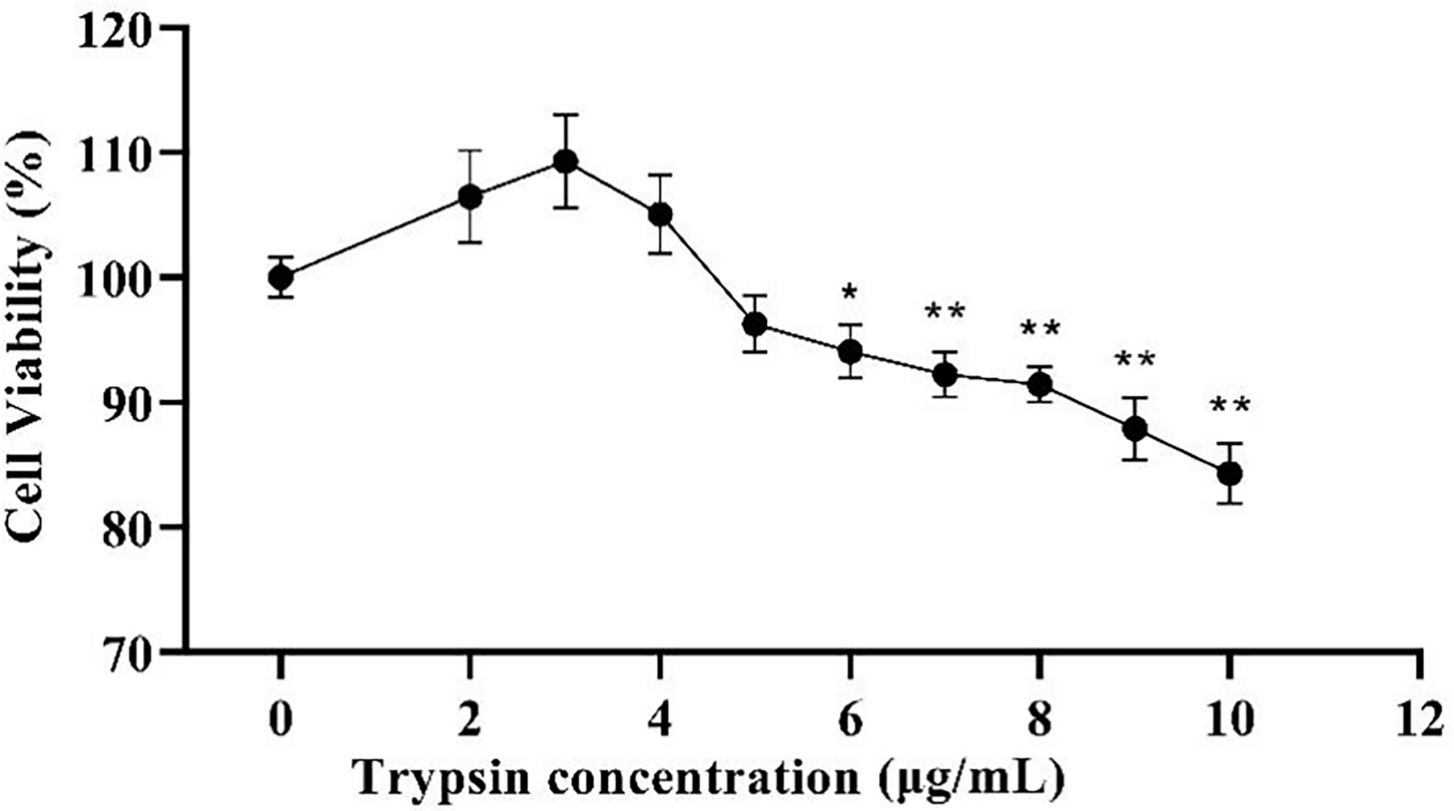
Effects of different trypsin concentrations on the viability of ST cells. * indicates that the difference is significant (*P* < 0.05) compared with the group added with 0 μg/mL trypsin, ** indicates that the difference is extremely significant (*P*<0.01), and no indication indicates that the difference is not significant (*P* > 0.05), n=6.

The addition of trypsin at ≤ 4 μg/mL had a non-significant promotion effect on ST cell viability compared to the control group (*P* > 0.05), while the addition of trypsin at ≥ 5 μg/mL inhibited the growth of ST cells, with a significant difference between 7 - 10 μg/mL of trypsin (*P* < 0.01) (**Figure 1**). Based on the results of the CCK-8 test, settings of 1, 2, 3, and 4 μg/mL of trypsin were used for subsequent tests.

**Figure 2:**
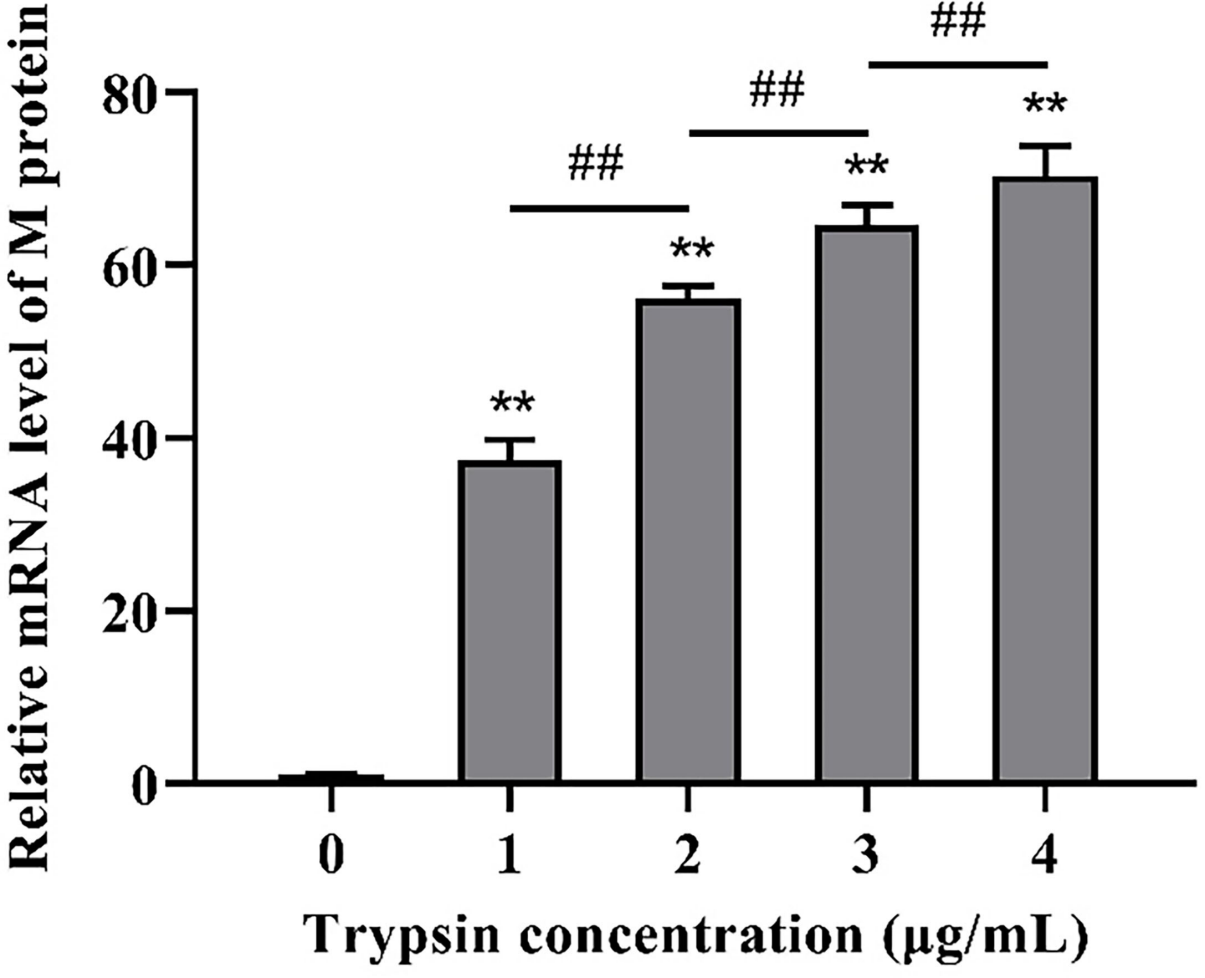
Effects of different trypsin concentrations on PDCoV replication. **indicates that the difference is extremely significant compared with the 0 μg/mL trypsin group (P < 0.01); ## indicates extremely significant difference (*P* < 0.01); No notation indicates that the difference is not significant (*P* > 0.05), n=6.

Following that, we looked at the expression of PDCoV M protein mRNA at various trypsin concentrations. As shown in **Figure 2**, in the concentration range of 1 - 4 μg/mL, viral replication increased significantly (*P* < 0.01) with the increase of trypsin concentration compared to the group without trypsin, in which the viral replication was highest after the addition of 4 μg/mL trypsin, about 70.4 times higher than that of the group without trypsin.

**Figure 3:**
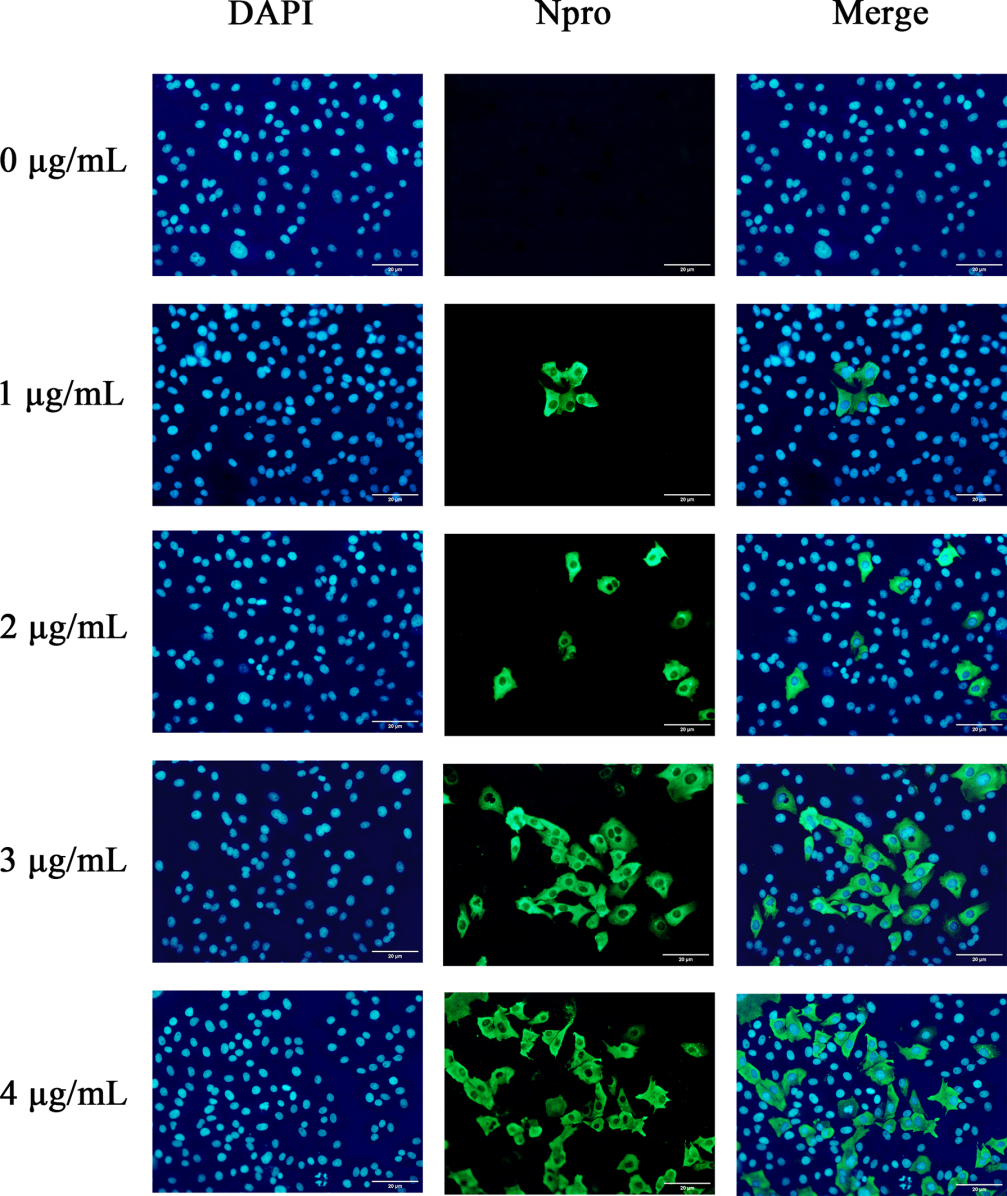
Changes in ST cells infected with PDCoV under different trypsin concentrations (400×). DAPI-stained nuclei are blue after UV light excitation and green after blue light excitation after primary and secondary antibodies binding to viral proteins.

To further confirm the optimal trypsin concentration for PDCoV infection of ST cells, we used the indirect immunofluorescence method to observe the infection of ST cells by PDCoV after adding different concentrations of trypsin. As shown in **Figure 3**, the cells with different concentrations of trypsin added could all observe obvious green fluorescence, whereas no fluorescence was observed in the group of cells without added trypsin; and the density of fluorescence increased with the increase of trypsin concentration in the range of 1 - 4 μg/mL, indicating that the higher the concentration of trypsin, the higher the expression of N protein, i.e., the more ST cells were infected with PDCoV. The density of cells infected with PDCoV was greatest at a trypsin concentration of 4 μg/mL, and therefore the addition of 4 μg/mL of trypsin was chosen for all subsequent experiments.

### Characterisation of SeNPs

**Figure 4:**
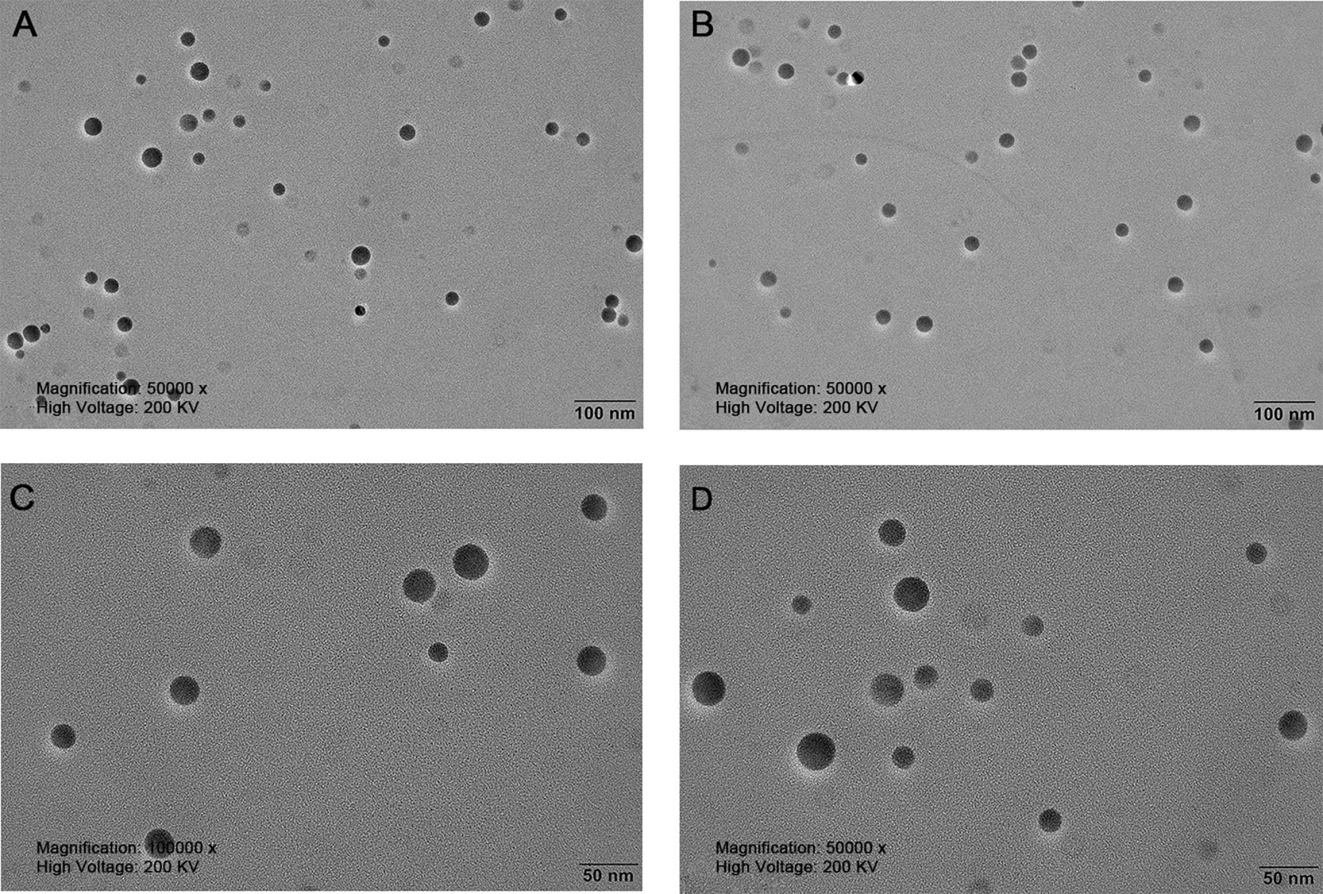
Transmission electron microscopy of SeNPs. **(A, B)** are magnified 50,000 times. **(C, D)** are magnified 100,000 times.

**Figure 5:**
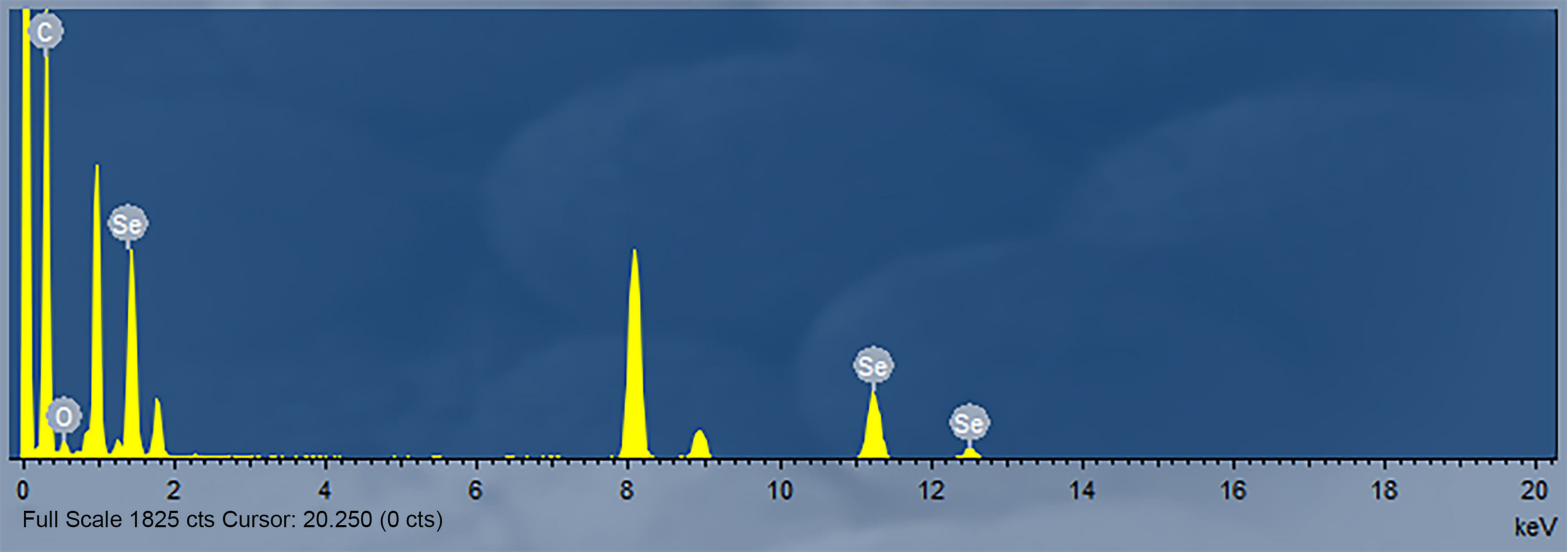
Energy Spectrum Analysis of SeNPs.

The nanoparticles were subsequently analyzed by EDX (**Figures 4**, **5**). The characteristic absorption peaks of SeNPs were present in the spectra at 1.37, 11.22, and 12.49 keV, respectively. in addition, signals of elements such as C and O were also present. the SeNPs The concentration of SeNPs was 2.8 mg/mL.

### Assay SeNPs on cell viability

**Figure 6:**
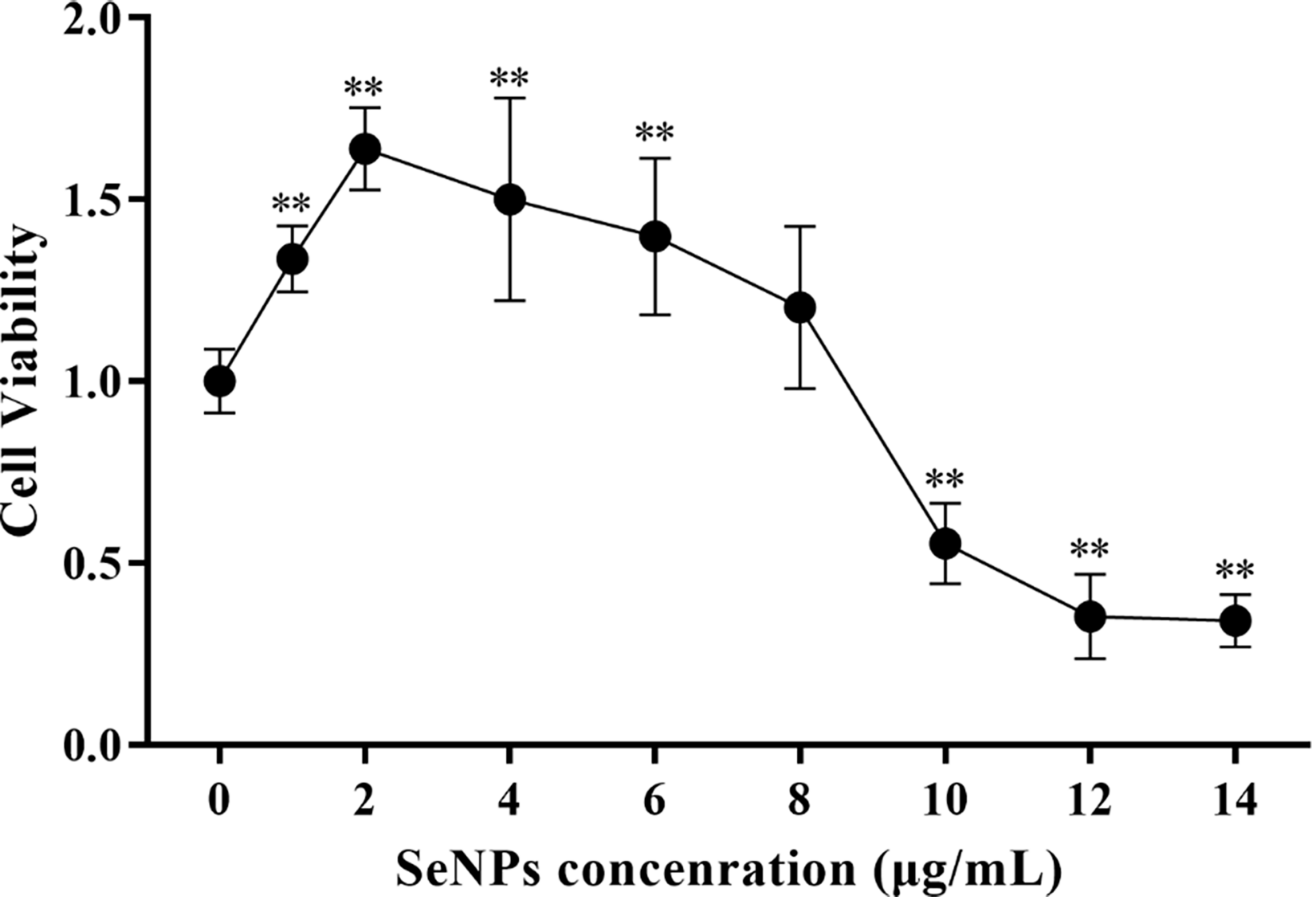
Effects of different SeNPs concentrations on the viability of ST cells In the figure ** indicates that the difference is extremely significant compared with the group added with 0 μg/mL SeNPs (P < 0.01), and no indication indicates that the difference is not significant (*P* > 0.05), n=6.

The cytotoxicity of SeNPs to ST cells in the range of 0 - 14 μg/mL (as determined by pre-experiment) was assessed using the CCK-8 method. As a result, SeNPs were not significantly cytotoxic in the range of 0 - 8 μg/mL, and the cell survival rate was greater than 100% (**Figure 6**). The data showed that the maximum concentration of SeNPs was 8 μg/mL. Based on this, 2, 4, and 8 μg/mL were selected for subsequent SeNPs antiviral assays.

### SeNPs inhibit the replication of PDCoV in ST cells

**Figure 7:**
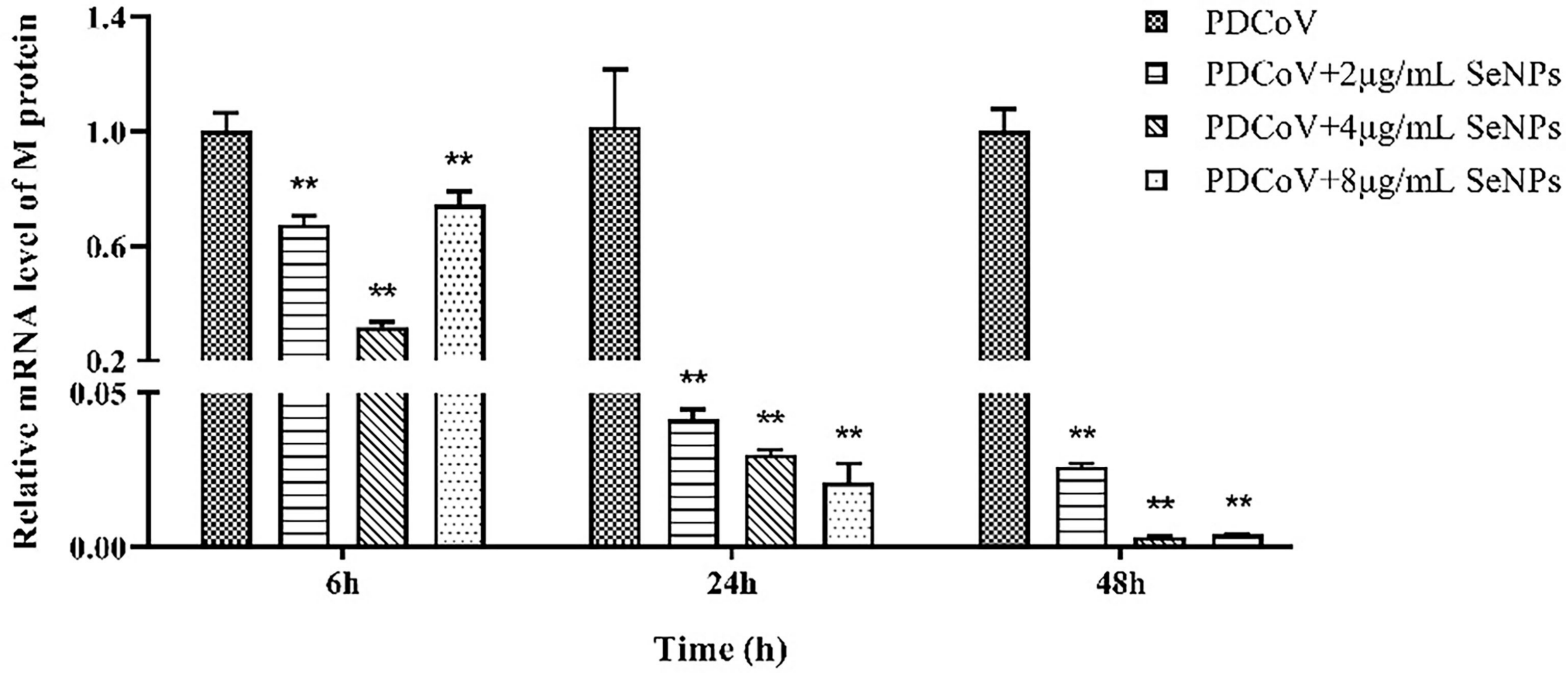
Effects of different concentrations of SeNPs on PDCoV replication in ST cells. ** indicates an extremely significant difference between different concentrations of SeNPs treatment group and PDCoV group (P <; 0.01), and no indication indicates no significant difference (*P* > 0.05), n=6.

PDCoV-infected ST cells were treated with 2, 4, and 8 μg/mL of SeNPs, and the expression of viral M protein mRNA was measured after 6, 24, and 48 h. As a result, all three concentrations of SeNPs inhibited viral replication significantly (*P* < 0.01) after 6 h of treatment, with the replication of 4 μg/mL SeNPs reaching approximately 0.318 times that of the non-SeNPs group (**Figure 7**). As the treatment time increased, the inhibitory effect of SeNPs became more significant, with the three concentrations at 24 h being 0.042, 0.030, and 0.021 times compared to the replication of the PDCoV groups, and at 48 h reaching 0.026, 0.003, and 0.004 times compared to the replication of the PDCoV groups, respectively. The above results demonstrate that SeNPs have a good anti-PDCoV effect and that this effect is enhanced with increasing duration of infection. Therefore, 4 μg/mL SeNPs were selected for subsequent experimental studies in this trial.

### SeNPs repress PDCoV-induced apoptosis in ST cells

**Figure 8:**
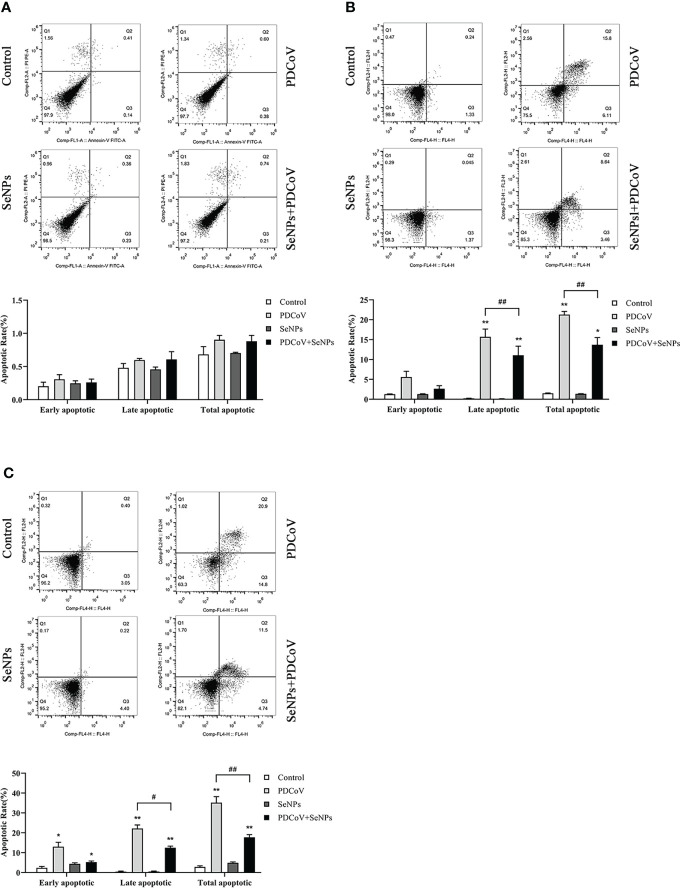
Effect of SeNPs on apoptosis induced by PDCoV. **(A–C)** are the flow quadrant diagram and apoptosis rate change diagram of each treatment group at 6, 24, and 48 h, respectively. * indicates that the difference is significant compared with the control group (*P* < 0.05), ** indicates that the difference is extremely significant compared with the control group (*P* < 0.01); # indicates that there is a significant difference between the PDCoV group and the PDCoV+SeNPs group (*P* < 0.05), ## indicates that there is a significant difference between the PDCoV group and the PDCoV+SeNPs group (*P* < 0.01), and there is no significant difference (*P* > 0.05) in the unlabeled expression, n=3.

It has been reported that PDCoV infection induces apoptosis in LLC-PK and ST cells. Our experiments demonstrated the inhibitory effect of SeNPs on PDCoV replication. To investigate whether SeNPs inhibit PDCoV-induced apoptosis, we measured the apoptosis rate by Annexin-FITC/PI double-staining assay after 6, 24, and 48 h treatment of infected cells with SeNPs. According to the findings in **Figure 8**, after 6h of PDCoV infection, the apoptosis rate increased slightly compared to the control group. There was no significant difference between the apoptosis rate in the SeNPs-treated group and the PDCoV group (*P* > 0.05), indicating that PDCoV infection had a lower effect on apoptosis in the early stage. The addition of SeNPs treatment did not inhibit the early infection-induced apoptosis few cells. With the increase in infection time, the late apoptosis rate, and total apoptosis rate were all significant increases in the PDCoV group compared with the control group after 24 h (*P* < 0.01). After SeNPs treatment of infected cells, the difference in early apoptosis rate was not significant (*P* > 0.05), late apoptosis and total apoptosis rates were significantly decreased *(P* < 0.01), and there was no significant change in the SeNPs group compared to the control group (*P* > 0.05). At 48h, compared to the controlgroup, the early apoptosis rate, late apoptosis rate and total apoptosis rateof the viral group were significantly increased (P < 0.01 or P < 0.05), and the late apoptosis rate and total apoptosis rate were significantly decreased after SeNPs treatment (P < 0.01). The above results indicated that PDCoV could induce apoptosis of ST cells *in vitro*, and the addition of SeNPs could significantly inhibit the apoptosis induced by virus. And the longer the infection time, the more the number of apoptotic cells, the more obvious the effect of its inhibition.

### SeNPs inhibit PDCoV-induced Cyt C release

**Figure 9:**
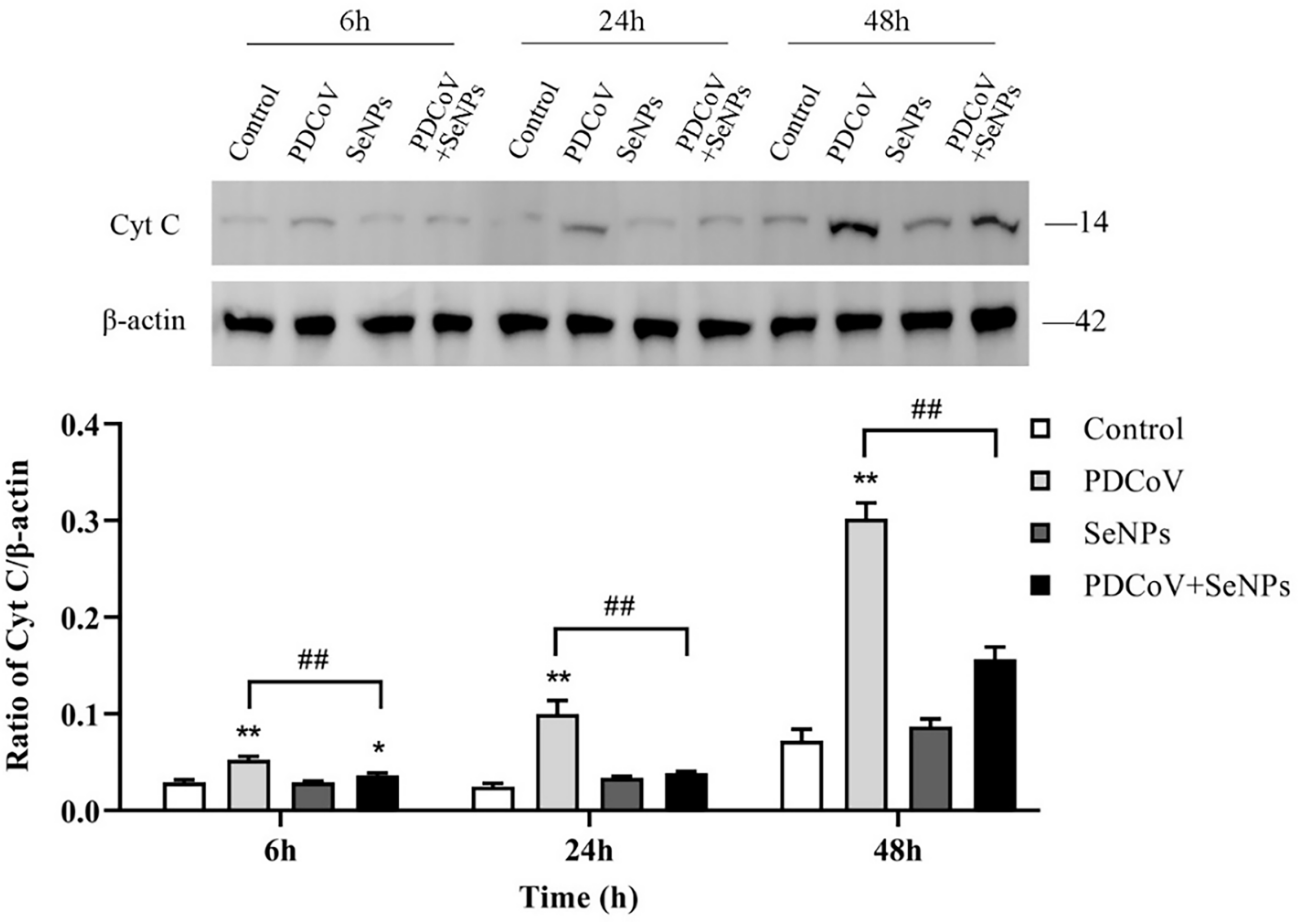
Effects of SeNPs on Cyt C content in the cytoplasm of ST cells infected with PDCoV. Top is immunoblot bands of Cyt C in each treatment group at 6, 24, and 48 h. At the bottom is a histogram after analyzing and calculating the ratio of grayscale values of Cyt C. * indicates that the difference is significant compared with the control group (P < 0.05), ** indicates that the difference is extremely significant compared with the control group (P < 0.01); ## indicates that there is a significant difference between the PDCoV group and the PDCoV + SeNPs group (P < 0.01), and there is no significant difference (P > 0.05) in the unlabeled expression, n=3.

Cyt C is an important apoptotic factor normally found in mitochondria and is released from mitochondria to the cytoplasm when apoptosis is stimulated, which is a critical event in apoptosis. The cytoplasmic Cyt C content was determined, and the results are shown in **Figure 9**. At the three time points, the control group had very few protein amounts of Cyt C in the cytoplasm, and the cytoplasm contained almost no Cyt C at 6 and 24 h, indicating that mitochondria release little or almost no Cyt C into the cells under normal conditions. In contrast, the cytoplasmic Cyt C contents were significantly higher in the PDCoV-infected groups at 6, 24, and 48 h than those in the control groups (*P* < 0.01) and increased with increasing infection duration, indicating that PDCoV infection could conduce the release of Cyt C from mitochondria, and the longer the duration of infection, the greater the amount of release. Cyt C content in the cytoplasm of the SeNPs-treated group (PDCoV+SeNPs) was significantly lower compared with the PDCoV group, and the differences were all highly significant (*P* < 0.01), while the SeNPs-treated group was significant (*P* < 0.05) at 6 h compared with the Control group. The SeNPs group was higher than the Control group at 6 h, 24 h and 48 h, but the differences were not significant (*P* > 0.05). The above results suggest that SeNPs treatment reduced Cyt C release, while the addition of SeNPs alone had no significant effect on Cyt C release.

### SeNPs inhibit PDCoV-induced Caspase 3 and 9 activation

**Figure 10:**
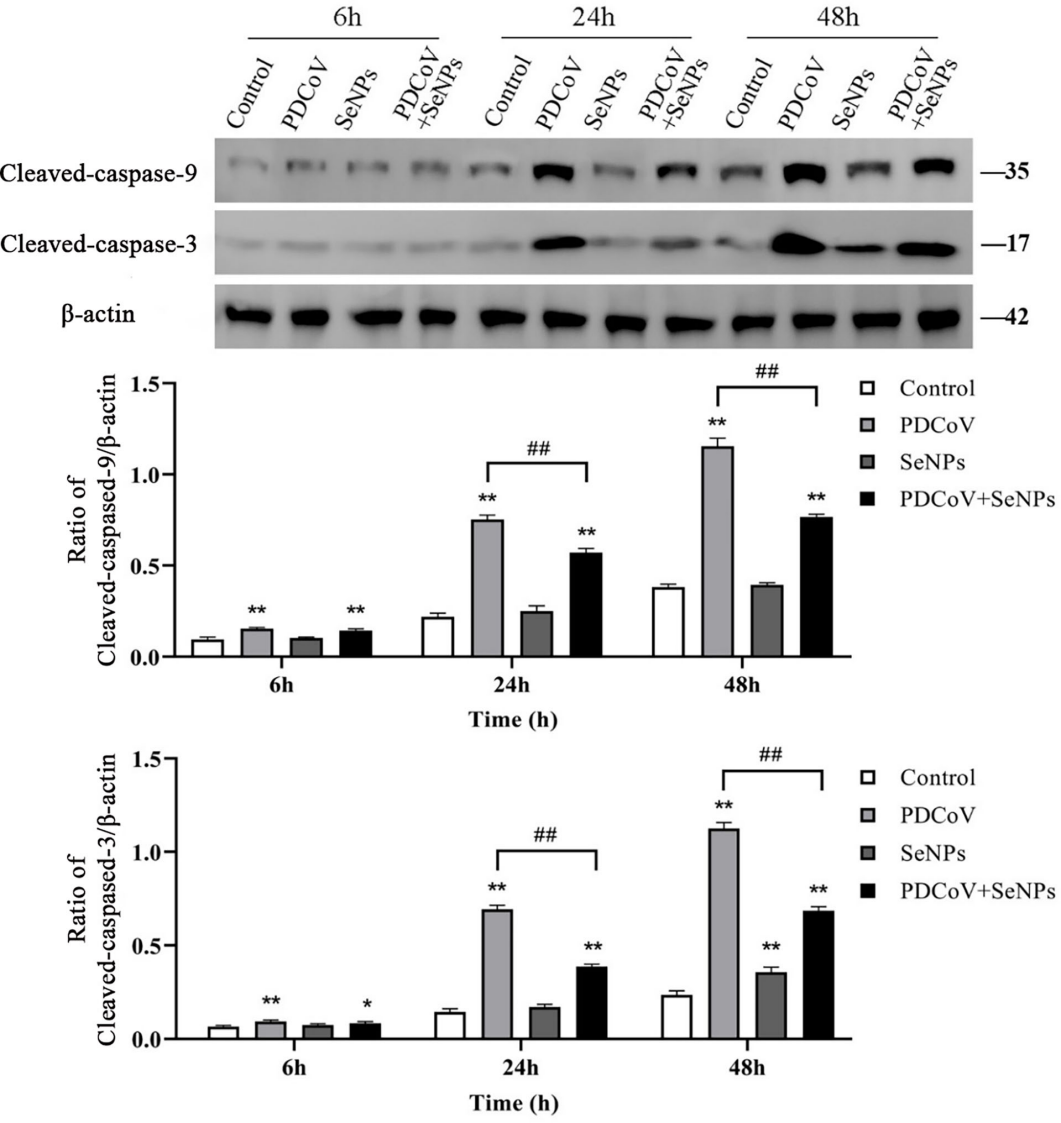
Effect of SeNPs on apoptosis-related proteins of ST cells infected with PDCoV. Top is immunoblot bands of Cleaved-caspase-3 and Cleaved-caspase-9 in each treatment group at 6, 24, and 48 h. At the bottom is a histogram after analyzing and calculating the ratio of grayscale values of the Cleaved-caspase-3 and Cleaved-caspase-9. * indicates that the difference is significant compared with the control group (P < 0.05), ** indicates that the difference is extremely significant compared with the control group (P < 0.01); ## indicates that there is a significant difference between the PDCoV group and the PDCoV + SeNPs group (P < 0.01), and there is no significant difference (P > 0.05) in the unlabeled expression, n=3.

After Cyt C is released into the cells, it activates downstream Caspase 9 and Caspase 3, further triggering the apoptotic cascade. To explore whether the two apoptotic proteins are activated after PDCoV infection and the effect of SeNPs treatment, we examined the activated Caspase 9 and Caspase 3 in the cells by the WB method, and the results are presented in **Figure 10**. The amount of activated Caspase 9 and Caspase 3 protein increased significantly after PDCoV infection compared to the control group, and the difference was significant (P < 0.01). And the longer the infection, the higher the expression of the two proteins. At 24 h and 48 h, after SeNPs were added, Caspase 9 protein content was lower than that of the PDCoV group, and the difference was significant (*P* < 0.01), and there was no significant change in protein content in the SeNPs group compared to the control group. Meanwhile, Caspase 3 levels were significantly lower in the SeNPs-treated group than in the PDCoV-infected group at 24 and 48 h (*P* < 0.01).

### SeNPs alleviate PDCoV-induced increased mitochondrial division

**Figure 11:**
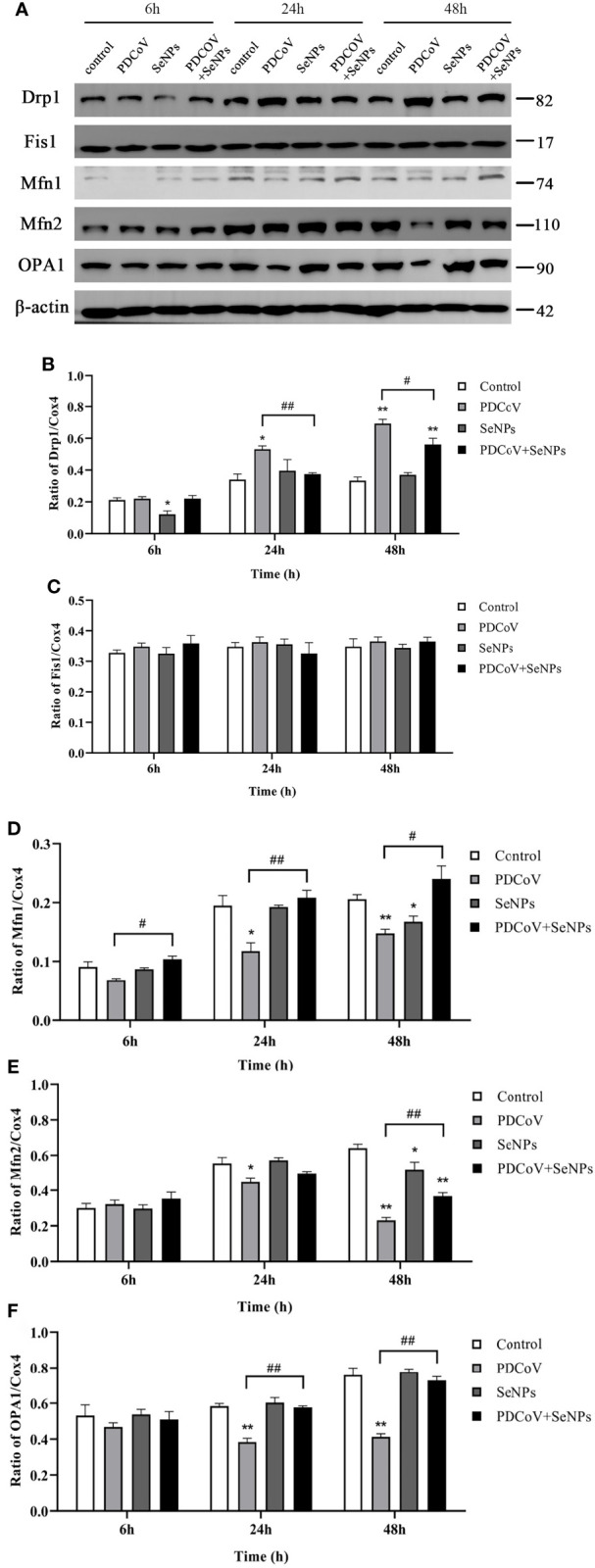
Effect of SeNPs on mitochondria dynamics-related proteins of ST cells infected with PDCoV. **(A)** Immunoblot bands of mitochondrial dynamic-related proteins Drp1, OPA1, Fis1, Mfn1, and Mfn2 in each treatment group at 6, 24, and 48 h; **(B–F)** are the histograms after analyzing and counting the gray value ratios of the target protein and the reference protein of Drp1, OPA1, Fis1, Mfn1, and Mfn2 respectively. * indicates that the difference is significant compared with the control group (P < 0.05), ** indicates that the difference is extremely significant compared with the control group (P < 0.01); # indicates that there is a significant difference between the PDCoV group and the PDCoV+SeNPs group (P < 0.05), ## indicates that there is a significant difference between the PDCoV group and the PDCoV+SeNPs group (P < 0.01), and there is no significant difference (P > 0.05) in the unlabeled expression, n=3.

To see if SeNPs’ inhibition of PDCoV-induced Cyt C release was associated with mitochondrial dynamics, we examined the changes in dynamics-related proteins in mitochondria after PDCoV infection and SeNPs treatment. The outcomes are depicted in **Figure 11**. Drp1 protein expression was significantly increased (*P* < 0.05 or *P* < 0.01) after 24h and 48 h PDCoV infection, but there was no effect on Fis1 protein expression. In contrast, the expression of mitochondrial fusion proteins Mfn1, Mfn2, and OPA1 significantly reduced after 24h and 48 h PDCoV infection (*P* < 0.05 or *P* < 0.01). According to the data presented above, PDCoV infection increases mitochondrial fission protein levels while decreasing fusion protein levels. And, within a certain range, the longer the infection time, the greater the amount of change. After adding SeNPs to infected cells, Drp1 protein levels reduced, while Mfn1, Mfn2, and OPA1 protein levels improved compared to the virus-infected group. In summary, PDCoV disrupted the dynamic balance of mitochondria in ST cells, resulting in increased mitochondrial division and decreased fusion, and SeNPs treatment can mitigate the excessive mitochondrial division to some extent.

## Discussion

PDCoV primarily affects the digestive tract of piglets *in vivo*, but studies have revealed that LLC-PK and ST cells are most susceptible to PDCoV *in vitro* (16) and that PDCoV can be isolated and passed continuously in these two cells, making them the most commonly used for PDCoV proliferation *in vitro*. Trypsin has been demonstrated to increase viral infectivity in cells (36). In a similar vein, after 24 h of incubation on ST cells without trypsin, no cells were infected with PDCoV by immunofluorescence, and when 4 μg/mL of trypsin was added, virus replication increased 70.4 times that of the non-addition group, which could be because trypsin promotes PDCoV attachment to cells (37) cleaving and activating the PDCoV S protein, allowing the virus to enter cells (38). Trypsin has also been proposed to promote viral replication by provoking the fusion of infected cells’ intercellular membranes (39).

In this experiment, we used an apoptosis assay to show that PDCoV caused a significant increase in the apoptosis rate of ST cells after 24 and 48 h of infection and that the longer the infection duration, the higher the apoptosis rate, indicating that PDCoV-induced apoptosis of ST cells in the middle and late stages of infection, and the induction of apoptosis became more obvious with the increase of infection duration, consistent with results by Jung et al. (1). Several studies showed that it promoted viral infection and replication by regulating apoptosis. The cytomegalovirus (CMV) viral protein vMIA blocks apoptosis by binding to the pro-apoptotic protein Bax, which is recruited to and bound to mitochondria. Rotavirus (RV) promotes the spread of virus progeny by activating the mitochondrial route of apoptosis in the late stages of infection, allowing viral particles to spread to surrounding cells (40, 41).

In most cell death models, the release of Cyt C from mitochondria results in the formation of an apoptosome with Apaf-1, which sequentially activates Caspase 9 and Caspase 3, triggering the apoptotic cascade response (23, 24). This experiment found that the amount of Cyt C protein in the cytoplasm significantly increased with time after PDCoV infection. Subsequent detection showed that the expression of Cyt C downstream proteins Caspase 9 and Caspase 3 increased after virus infection, and the content was positively correlated with the infection time, indicating that PDCoV induces the mitochondrial release of Cyt C and then activates the Caspase apoptosis cascade. Mitochondrial dynamics are tightly linked to apoptosis. Many studies have argued that increased mitochondrial division leads to apoptosis, and many viruses take advantage of this to disrupt the dynamics balance to induce apoptosis (42). Therefore, we investigated whether PDCoV disrupts mitochondrial morphology by interfering with mitochondrial dynamics, further leading to the release of Cyt C. After PDCoV infection, we identified a substantial increase in the mitochondrial fission protein Drp1 and a decrease in the expression of the fusion proteins Mfn1, Mfn2, and OPA1, indicating that the virus causes excessive mitochondrial division. Similar to the results of this assay, Mukherjee et al. discovered that RV infection of cells resulted in increased mitochondrial division, the release of Cyt C, and consequent activation of the apoptotic pathway by promoting the expression of the fission protein Drp1 and degradation of the fusion protein Mfn1 (41). It has also been demonstrated that Drp1-mediated mitochondrial division induces the release of Cyt C. When Drp1 expression is inhibited, it reduces fragmented mitochondria and inhibits Cyt C release and nuclear DNA fragmentation (43). OPA1 is a mitochondrial cristae remodeling protein stabilizing cristae morphology and limiting intracristae protein release. The significant decrease in OPA1 expression observed in this experiment could also be a crucial factor in the release of Cyt C from mitochondrial cristae. The relationship between mitochondrial division and apoptosis is still being debated. Although mitochondrial division is commonly thought to be an early event in apoptosis, some studies have found that inhibiting mitochondrial division only had a minor effect on Cyt C release and had no effect on the release of other apoptotic factors in mitochondria, implying that mitochondrial division may not be the primary cause of Cyt C release (44). It has also been argued that mitochondrial division is only essential when large amounts of Cyt C are required to activate the Caspase pathway (45). Hence more studies are needed to clarify the relationship between the two in the future.

In our another experiment, ST cells were treated by four kinds of interactions between SeNPs and virus *in vitro*. Samples were collected to detect the amount of virus replication in cells to explore the antiviral effect of SeNPs. The experiment contains anti-adsorption effect of SeNPs (equal volumes of virus and SeNPs were added directly to the cells, incubated for 1 h at 4°C refrigerator, washed, added to trypsin culture medium, and incubated in the cell incubator), preventive effect of SeNPs (virus was added to the cells pretreated with SeNPs for 1 h, incubated for 1 h in the cell incubator, washed, added to culture medium, and incubated in the cell incubator), therapeutic of SeNPs (virus pretreated cells, incubated in the cell incubator for 1 h, washed, added to the culture medium containing trypsin and SeNPs and placed in the cell incubator), and direct inactivation of SeNPs (virus and SeNPs mixture preincubated at 4°C for 1 h, incubated in the cell incubator for 1 h, washed, added to the culture medium and placed in the cell incubator). It was found (data not shown in this paper) that direct inactivation of the virus by SeNPs was nor significant. And the optimal effect related to SeNPs is therapeutic effect compared to anti-PDCoV adhesion effect, protective effect, direct inactivation effect. Therefore, we suggest that SeNPs inhibits apoptosis caused by viruses by alleviating excessive mitochondrial division, but deeper mechanism needs further trials to verify.

There are no effective drugs for PDCoV treatment or prevention. Although many studies have shown that certain drugs can inhibit PDCoV replication (46–49), these drugs are not used in production. As a result, finding an anti-PDCoV drug that can be applied in production is a problem that must be solved. In this study, we discovered that the viral M-gene replication reached 0.318, 0.030, and 0.003 times respectively compared to the control groups at 6, 24, and 48 h after adding 4 μg/mL of SeNPs, indicating that SeNPs had a strong anti-PDCoV ability. The inhibition of virus replication by selenium *in vitro* has been reported in various reports. Diphenyl diselenide (PhSe) inhibited the replication of herpes simplex virus 2 (HSV-2) in Vero cells (50). Na_2_SeO_3_ inhibited the replication of the hepatitis B virus (HBV), and the inhibitory effect increased with increasing Na_2_SeO_3_ concentration or treatment time (51). In Madin-Darby Canine Kidney (MDCK) cells, modified SeNPs inhibit the H1N1 influenza virus and Caspase 3-mediated apoptosis caused by virus infection (52, 53). Our previous research found that selenomethionine (Se-Met) inhibited PDCoV replication on LLC-PK cells (29). However, most of the above studies are on inorganic and organic selenium, and there are few studies on the antiviral effects of SeNPs. We compared the results of this trial to the previous ones and discovered that SeNPs had better anti-PDCoV effects than Se-Met, which could be due to SeNPs’ easier absorption, implying that SeNPs have greater antiviral potential than organic and inorganic selenium and could be a viable drug for the treatment of PDCoV and other virus infections.

Current studies associated selenium are primarily focused on its antiviral and antioxidant properties, with few reports linking selenium to apoptosis. Some studies have shown that selenium can inhibit apoptosis caused by certain toxic agents. Wang et al. discovered that L-selenomethionine reduced excessive apoptosis induced by Ammonia (NH3) and abnormal changes in mitochondrial dynamics-related proteins (54). Selenium-rich yeast (SeY) attenuated potassium dichromate (K_2_Cr_2_O_7_) induced apoptosis in poultry kidney tissue *via* modulating the mitogen-activated protein kinase pathway (55). Wang et al. manifested that sodium selenite (SS) and (Se-Met) could reduce tertbutyl hydroperoxide (TBHP) induced oxidative stress, inhibited mitochondrial fission and apoptosis of nucleus pulposus cells (NPCs) (56). *In vitro* studies have shown that selenium supplementation can inhibit viral infection by increasing glutathione peroxidase 1 (Gpx1) activity and reducing reactive oxygen species (ROS) content, JNK phosphorylation levels (57–59).

According to our findings, adding SeNPs to infected cells significantly reduced the apoptosis rate and inhibited the release of Cyt C and the activation of Caspase 9 and Caspase 3, implying that SeNPs inhibit PDCoV-induced apoptosis. Subsequent experiments revealed that SeNPs could, to some extent, reduce the increase in Drp1 protein level caused by PDCoV and elevate the decrease in Mfn1, Mfn2, and OPA1 protein levels caused by PDCoV. In summary, it may be inferred that SeNPs exerted antiviral effects by alleviating excessive mitochondrial division and inhibiting Cyt C release in the middle and late stages of PDCoV infection, thereby antagonizing virus-induced apoptosis and thus inhibiting the propagation and spread of virus particles, but more studies are needed to confirm this point.

## Data availability statement

The original contributions presented in the study are included in the article/supplementary materials. Further inquiries can be directed to the corresponding author.

## Ethics statement

Ethical review and approval was not required for the study on human participants in accordance with the local legislation and institutional requirements. Written informed consent from the patients/participants OR patients/participants legal guardian/next of kin was not required to participate in this study in accordance with the national legislation and the institutional requirements.

## Author contributions

ZR, ZW, and HH contributed to the conception and design of the study. ZR, YY, XZ, QW performed the experiments. CC, JD, ZW, RS performed statistical analysis. YY and RS wrote the first draft of the manuscript. The rest reviewed and revised the manuscript. All authors reviewed the manuscript, read and approved the submitted version.

## Funding

This study was supported by the National Key Research and Development Program of China (2021YFD1801103-4), and the National Natural Science Foundation of China (32130106).

## Conflict of interest

The authors declare that the research was conducted in the absence of any commercial or financial relationships that could be construed as a potential conflict of interest.

## Publisher’s note

All claims expressed in this article are solely those of the authors and do not necessarily represent those of their affiliated organizations, or those of the publisher, the editors and the reviewers. Any product that may be evaluated in this article, or claim that may be made by its manufacturer, is not guaranteed or endorsed by the publisher.

## Abbreviations

Caspase, Cysteinyl aspartate specific proteinase; Cyt C, Cytochrome C; Drp1, Dynamin-like protein1; Fis1, Mitochondrial fission protein1; LLC-PK, LLC porcine kidney cell line; Mfn1/2, Mitofusin1/2; OPA1, Optic Atrophy1; PDCoV, Porcine deltacoronavirus; qPCR, Quantitative Real-time PCR; SeNPs, Selenium Nano-Particles; ST, Swine testis; WB, Western Blotting.
